# Existing plaques and neuritic abnormalities in APP:PS1 mice are not affected by administration of the gamma-secretase inhibitor LY-411575

**DOI:** 10.1186/1750-1326-4-19

**Published:** 2009-05-06

**Authors:** Monica Garcia-Alloza, Meenakshi Subramanian, Diana Thyssen, Laura A Borrelli, Abdul Fauq, Pritam Das, Todd E Golde, Bradley T Hyman, Brian J Bacskai

**Affiliations:** 1 MassGeneral Institute for Neurodegenerative Diseases, Massachusetts General Hospital, 114 16th Street, Charlestown, MA 02129, USA; 2 Area de Fisiología, Facultad de Medicina, Universidad de Cádiz, Plaza de Falla 9. 11003 Cádiz, Spain; 3 Department of Neuroscience, Mayo Clinic Jacksonville, Birdsall 210, 4500 San Pablo Rd, Jacksonville, FL 32224, USA

## Abstract

The γ-secretase complex is a major therapeutic target for the prevention and treatment of Alzheimer's disease. Previous studies have shown that treatment of young APP mice with specific inhibitors of γ-secretase prevented formation of new plaques. It has not yet been shown directly whether existing plaques would be affected by γ-secretase inhibitor treatment. Similarly, alterations in neuronal morphology in the immediate vicinity of plaques represent a plaque-specific neurotoxic effect. Reversal of these alterations is an important endpoint of successful therapy whether or not a treatment affects plaque size. In the present study we used longitudinal imaging in vivo with multiphoton microscopy to study the effects of the orally active γ-secretase inhibitor LY-411575 in 10–11 month old APP:PS1 mice with established amyloid pathology and neuritic abnormalities. Neurons expressed YFP allowing fluorescent detection of morphology whereas plaques were labelled with methoxy-XO4. The same identified neurites and plaques were followed in weekly imaging sessions in living mice treated daily (5 mg/kg) for 3 weeks with the compound. Although LY-411575 reduced Aβ levels in plasma and brain, it did not have an effect on the size of existing plaques. There was also no effect on the abnormal neuritic curvature near plaques, or the dystrophies in very close proximity to senile plaques. Our results suggest that therapeutics aimed at inhibition of Aβ generation are less effective for reversal of existing plaques than for prevention of new plaque formation and have no effect on the plaque-mediated neuritic abnormalities, at least under these conditions where Aβ production is suppressed but not completely blocked. Therefore, a combination therapy of Aβ suppression with agents that increase clearance of amyloid and/or prevent neurotoxicity might be needed for a more effective treatment in patients with pre-existing pathology.

## Background

Alzheimer's disease (AD) is the most common cause of dementia among elderly people and it has no known cure. Compelling evidence from histological and biochemical studies support the idea that the accumulation of amyloid-β (Aβ) aggregates in the brain plays a seminal role in the pathogenesis of AD [1]. Likewise, the genetic evidence regarding familial mutations of the amyloid precursor protein (APP) and presenilins support the pathogenic role of Aβ accumulation [2]. Aβ deposits as compact or dense core plaques that are sources of focal neurotoxicity in transgenic mice and in AD [3]. In this regard, senile plaques are associated with neuritic dystrophies and synaptic loss [4-6] and it has also been shown that senile plaques may disrupt cortical synaptic integration[7].

Aβ is generated after sequential cleavage of APP by β and γ-secretases. Therefore, both β-secretase [8] and γ-secretase inhibitors are primary pharmacological targets in the treatment of AD (for review see [9-11]). The γ-secretase complex is constituted by at least four integral membrane proteins including presenilin, nicastrin, APH-1 and PEN-2. The activity of γ-secretase determines the solubility of the Aβ fragments, with Aβ42 more prone to aggregation than the shorter cleavage products [10]. Due to these considerations, different approaches towards modulating γ-secretase activity towards producing shorter peptide fragments are being developed. There has been considerable success in generating small molecules capable of entering the central nervous system that inhibit γ-secretase activity potently leading to a sustained reduction in brain Aβ levels [12]. In both humans and animal models, the use of γ-secretase inhibitors to reduce Aβ levels and slow Aβ deposition has been demonstrated. Administration of γ-secretase inhibitors significantly reduced Aβ levels in plasma in control and AD patients [13,14], as well as in CSF [15]. Similarly, it has also been shown that inhibiting γ-secretase activity can reduce Aβ levels in plasma, CSF and brain both in young and aged transgenic mice [16-18] and long-term treatments can slow senile plaque deposition in Tg2576 mice [19]. Moreover, acute treatment with γ-secretase inhibitors led to partial reversal of the deficits in hippocampal-dependent contextual fear conditioning test in Tg2576 mice [20]. The previous work has demonstrated positive effects of γ-secretase inhibition therapy to prevent or slow Aβ progression. It is unknown, however, whether inhibiting γ-secretase activity will be effective in a treatment paradigm. Will inhibition of γ-secretase lead to the clearance of existing plaques or the reversal of the morphological alterations in neurons in the mouse models of AD? In the present work, we use a well characterized γ-secretase inhibitor, N(2)-[(2S)-2-(3,5-difluorophenyl)-2-hydroxyethanoyl]-N(1)-[(7S)-5-methyl-6-oxo-6,7-dihydro-5H-dibenzo[b,d]azepin-7-yl]-l-alaninamide (LY-411575) [21,22] and multiphoton microscopy to assess *in vivo *the effect of long-term treatment on existing senile plaques and the neuronal abnormalities associated with the plaques in APPswe/PS1dE9 mice. This animal model shows early deposition of Aβ by 4–6 months of age [23,24] and develops neuritic dystrophies and abnormal neuritic curvature [5,25]. Therefore, at the age used in this study (10–11 months old) the Aβ deposition and related neuropathological changes represent a model of established neuropathology.

## Results

### *In vivo *effect of LY-411575 on size of existing plaques

The orally active γ-secretase inhibitor LY-411575 was administered daily via gavage for 3 weeks to APP:PS1 mice with pre-existing amyloid pathology. No adverse effects were observed in the mice during this treatment period. Using longitudinal imaging of the brain with multiphoton microscopy, the size of individual, identified plaques was monitored.

**Figure 1:**
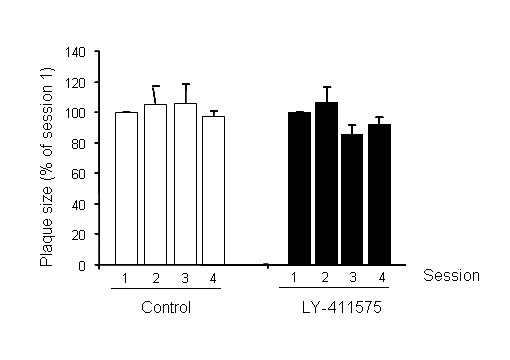
**LY-411575 treatment had no effect on the size of individual plaques in APPswe/PS1dE9xYFP mice**. Individual, identified plaques were monitored with longitudinal imaging during the course of the treatment using multiphoton microscopy. Plaques in control treated animals did not change in size, as previously described [25,26]. Existing plaques in the treated mice were also unaffected; we did not detect a treatmentXsession effect [F_(3,218) _= 1.087; P = 0.356] or a treatment effect. However, there was a trend towards a slight reduction in plaque size at 2 weeks that was maintained until the end of the treatment. Data are representative of 16–57 plaques from 6–7 animals.

Mice were injected with methoxy-XO4 to label amyloid pathology and fluorescent angiograms were used to identify imaging volumes over time. With each plaque serving as its own control, as previously described [26], there was no significant effect of LY-411575 treatment on plaque size throughout the treatment period (Figure 1). This result suggests that prevention of amyloid production with potent secretase inhibitors has no effect on the size of existing plaques over a 3 week treatment protocol. It should be noted, however, that this may be a limitation of the dosage or treatment duration. Likewise, our measurements were based on using methoxy-XO4 as the plaque label, and this reports the congophilic core of plaques, not the halo of immuno-positive amyloid that tends to surround individual plaques. Hence, it is possible that very small or undetectable effects on plaque size may still lead to reduced neurotoxicity and beneficial effects.

### *In vivo *effect of LY-411575 on neuritic abnormalities

**Figure 2:**
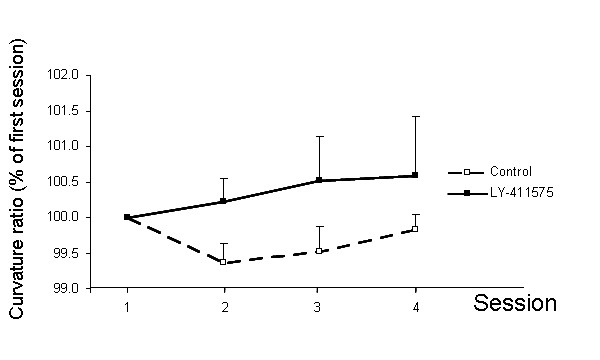
**LY-411575 treatment had no effect on neuritic curvature in APPswe/PS1dE9xYFP mice when neurites up to 50 μm from the plaque border were analyzed**. Data are representative of 44–138 neurites from 8–9 animals. The first imaging session (session 1) was acquired before the commencement of the treatment. Animals received daily gavage administrations of LY-411575 (5 mg-Kg) or vehicle and for the next 3 weeks and imaging sessions were completed on a weekly basis (sessions 2, 3 and 4). The curvature ratio of the same identified neurites were measured over time, with each neurite serving as its own control. No treatmentXsession effect was detected [F_(3,561) _= 0.171; P = 0.916] despite a slight trend towards a straightening effect observed in LY-411575 mice at the conclusion of the treatment.

**Figure 3:**
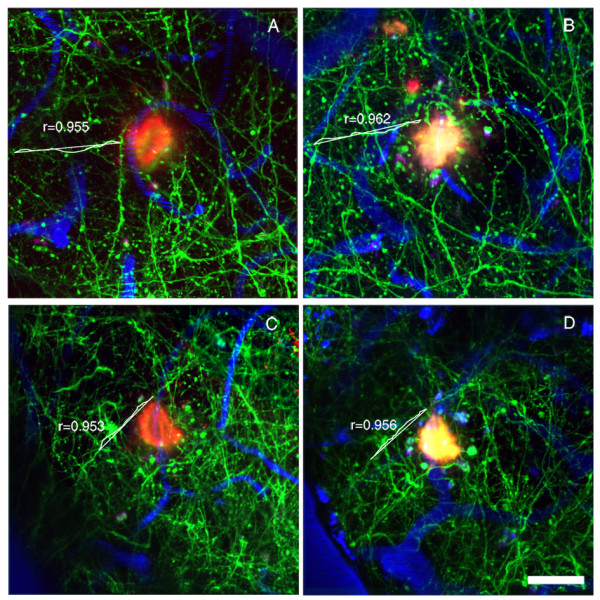
**Representative example of the effect of LY-411575 treatment on neuritic curvature in APPswe/PS1dE9xYFP mice**. Animals received daily oral administration of LY-411575 (5 mg/Kg) or vehicle for 3 weeks. These *in vivo *multiphoton images were taken before starting the treatment (session 1, A-LY-411575, C-vehicle) and after 3 weeks of treatment (session 4, B-LY-411575 and D-vehicle). Each image is a maximum intensity projection of a small volume of the brain. Individual neurites are traced for the curvature measurements before and after treatment. Neurons are green, blood vessels are blue, and dense-core Aβ plaques (red) were labeled by systemic injection of methoxy-XO4. Scale bar: 25 μm

It has been shown previously that neuritic processes in the vicinity of senile plaques are significantly more distorted, or "curvy", supporting the toxic effect of senile plaques and providing a useful model to study neuronal pathophysiology [5,25]. Using the quantitative curvature ratio, we have previously shown that these neuritic abnormalities can be at least partially reversed in short periods of time with candidate therapeutics [5,6,27]. We monitored these pathological abnormalities through the use of APPswe/PS1dE9 mice crossed with neuron specific YFP expression [28] to allow simultaneous multiphoton imaging of Aβ deposits and neuronal processes throughout the treatment period. We examined the neurites located in the close proximity to senile plaques (within 50 μm of plaque borders) where the distorted pathology is most severe. The average distance of the measured neurites to plaques before the beginning of the treatment for Control mice was 21.0 ± 1.5 μm (n = 66), and 24.1 ± 1.2 μm (n = 101) for LY-411575 treated mice. No statistical differences between average distances to plaques were detected using Student-t test (P = 0.110). The stability of the selected population was also assessed in the consecutive sessions and no treatmentXsession effect was detected using two way ANOVA for repeated measures ([F_(3,306) _= 0.311; P = 0.817] n = 11–101 from 3–4 mice), indicating that the selected neuritic populations were comparable. We did not observe any effect of the γ-secretase inhibitor LY-411575 on the shaft diameter of the selected neurites (table 1). When we examined the morphology of the selected neurites, we observed a curvature ratio before starting the treatment (session 1, day 0) in the range previously described for this model (~0.955) [5,25] both for Control and LY-411575 groups. We did not detect a treatmentXsession effect using two-way ANOVA for repeated measures after 3 weeks of oral administration of LY-411575 (Figure 2, Figure 3 for representative example).

**Table 1:** LY-411575 has no effect on dystrophy size or neurite diameter in APPswe/PS1dE9xYFP mice

**Treatment**	**Dystrophy size (μm^2^)**		**Neurite diameter (μm)**	
	**Control**	**LY-411575**	**Control**	**LY-411575**

**Session 1**	5.4 ± 0.7	4.7 ± 0.3	0.95 ± 0.04	0.97 ± 0.02

**Session 2**	4.3 ± 0.9	5.5 ± 0.5	0.95 ± 0.03	1.07 ± 0.05

**Session 3**	6.3 ± 0.9	6.5 ± 0.5	1.00 ± 0.04	1.12 ± 0.05

**Session 4**	4.3 ± 0.5	6.2 ± 0.5	0.99 ± 0.05	1.12 ± 0.06

LY-411575 (5 mg/Kg) had no effect on dystrophy size when dystrophies located in close proximity to senile plaques (within 15 μm) were measured in four consecutive imaging sessions using multiphoton imaging. The first imaging session (session 1) was acquired before the commencement of the treatment. Animals received daily gavage administrations of LY-411575 or vehicle for 3 weeks. Imaging was performed on a weekly basis (sessions 2, 3 and 4). Analysis using a two way ANOVA for repeated measures resulted in: sessionXtreatment [F_(3,1968) _= 1.461; P = 0.223]). Data are from 3–4 mice and 36–534 dystrophies. We also did not detect any significant effect on shaft diameter of the neurites, located within 50 μm of plaques, (two way ANOVA for repeated measures sessionXtreatment [F_(3,329) _= 0.884; P = 0.450]). Data are from 3–4 mice and 23–111 neurites.

We also monitored an additional neuritic alteration comprised of dystrophic swellings of neurites in the immediate vicinity of plaques. These dystrophies, which are thought to be dendritic as well as axonal [29], represent a distinct manifestation of plaque-specific neurotoxicity that can be at least partially reversed with candidate therapeutics [27,30]. When we assessed the size of these dystrophic swellings over time, we did not observe any effect after 3 weeks of treatment. Similarly, when we normalized the dystrophy size to plaque size we did not detect a significant sessionXtreatment effect [F_(3,129) _= 0.428; P = 0.734] (data not shown). These results demonstrate that this plaque associated neuronal pathology was not improved with treatment with the γ-secretase inhibitor. All together, these results suggest that even after 3 weeks of treatment, there was no detectable effect of γ-secretase inhibition on the neurotoxicity, manifested as morphological changes in neuronal processes, associated with existing plaques in vivo.

### Effect of LY-411575 on Aβ40 and 42 levels in plasma

**Figure 4:**
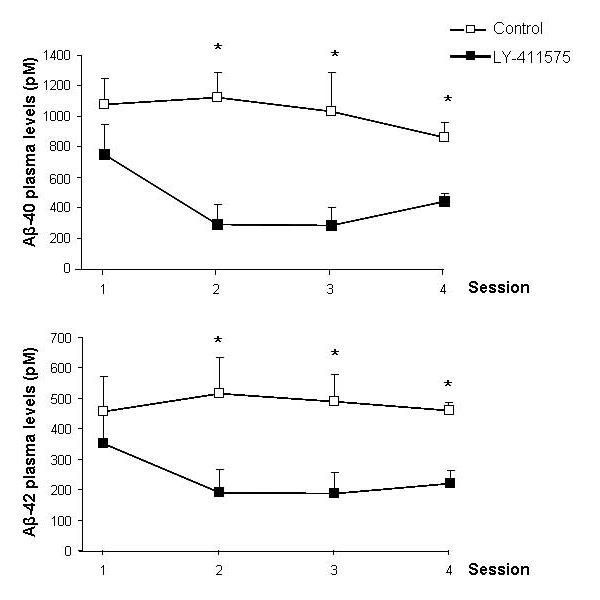
**LY-411575 treatment reduced Aβ40 and 42 plasma levels in APPswe/PS1dE9xYFP mice**. Animals received daily oral administration of LY-411575 (5 mg/Kg) or vehicle for 3 weeks. Plasma samples were taken before starting the treatment (session 1) and on a weekly basis for the next 3 weeks. All measurements were done in duplicate and data are representative of 3–5 mice. We observed a significant sessionXtreatment effect [F_(2,14) _= 6.307; P = 0.011] and [F_(2,14) _= 11.806; P = 0.001] for Aβ40 and 42 respectively. Student-t test for independent sessions showed significant differences for both measurements after the first week of treatment when compared with control values. Differences were maintained until the end of the treatment (*p < 0.05).

Blood samples were taken at weekly intervals during the course of the study to monitor the effectiveness of γ-secretase inhibition. A standard ELISA kit was used to determine soluble Aβ40 and 42 levels over time. Plasma levels of both Aβ40 and 42 were significantly reduced in LY-411575 treated mice within one week after the commencement of the treatment when compared to Control values, and this effect was maintained until the end of the treatment (Figure 4). This result demonstrates that the dose of inhibitor used was able to reduce circulating Aβ levels by approximately 60% throughout the course of treatment.

### Effect of LY-411575 on Aβ40 and 42 levels in brain

**Figure 5:**
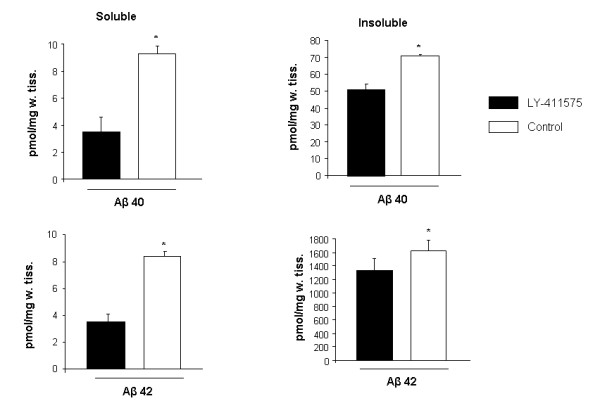
**LY-411575 treatment reduced soluble and insoluble Aβ40 and 42 brain levels in APPswe/PS1dE9xYFP mice**. All measurements were done in triplicate and data are representative of 4–6 mice. Student-t test for independent samples showed a significant reduction of soluble and insoluble Aβ40 and 42 brain levels after 3 weeks of treatment when compared with control values (*p < 0.05).

Biochemical measures of the effectiveness of the treatment were also used. At the end of the experiments, mice were killed, the brains extracted, and one hemisphere was flash frozen. These samples were homogenized and analyzed for Aβ levels using standardized ELISA kits. The levels of soluble Aβ40 and 42 in the brain were significantly reduced at the end of the treatment when compared to control values (Figure 5), supporting previous studies where similar effects were reported in a different mouse model [22]. The levels of formic acid extractable (insoluble) Aβ40 and 42 were also reduced, but to a lesser extent. These results demonstrate that LY-411575 can reduce total Aβ levels in the brain within this 3 week treatment period, in accord with previous reports.

## Discussion

It is well established that exaggerated production and deposition of Aβ aggregates as soluble oligomers, ADDLs, and senile plaques plays a key role in the pathogenesis of AD [31,32]. Therefore, blocking the production, limiting aggregation, or enhancing Aβ clearance has become a major challenge in the treatment of the illness [33]. Concerted efforts have been directed towards the prevention of production of Aβ through inhibition of γ-secretase. The γ-secretase is a multi-component protease complex that catalyzes the intramembranous cleavage of a number of type I tansmembrane proteins, including APP. Inhibiting APP processing reduces Aβ production and this may prevent or limit the progression of the illness. Although an extensive bibliography supports the capacity of γ-secretase inhibitors to prevent Aβ formation and aggregation both in human and in transgenic mouse models [2,13,17,22], it remains unclear whether γ-secretase inhibition can also lead to reversal of existing pathology or the associated neuropathology. This distinction is important in the context of preventative vs treatment paradigms.

In the present work, we used as proof-in-principle the low molecular weight γ-secretase inhibitor LY-411575; a highly potent compound that crosses the blood-brain barrier and interacts with a binding site located in the PS1 C-terminal fragment [34]. It is well established that γ-secretase also leads to cleavage of other substrates, with Notch being an important alternative substrate. It has been reported, for example, that Notch inhibition induced by γ-secretase inhibitors can reduce thymus size and induce hyperplasia in the gastrointestinal track, however these side effects were reversible and can be controlled by adjusting dosage and length of the treatment [21]. Although we did not specifically check for adverse gastrointestinal and thymus effects, the dose used in this study (5 mg/Kg/day) did not induce the "sickly" phenotype described by Hyde et al [21] at higher doses.

We did, however, observe that chronic γ-secretase inhibition significantly reduced Aβ40 and 42 plasma levels within 1 week after the commencement of the treatment. This effect confirms previous studies in other transgenic mouse models [12,16,20,22,35] and in non transgenic mice [18]. Moreover, γ-secretase inhibitors have been shown to reduce Aβ40 and 42 plasma levels both in healthy volunteers [2,13] and in AD patients [14]. Similarly, when we assessed brain Aβ40 and Aβ42 levels in APPswe/PS1dE9 mice we observed a significant effect after 3 weeks of treatment as previously shown with this compound in other transgenic mice [19,22,35]. All together, these data further support the capacity of LY-411575 to cross the blood-brain barrier and reduce Aβ levels both centrally and systemically. However, when we assessed the effect of LY-411575 on existing senile plaques we observed no reduction of plaque size after 3 weeks of treatment. Identified plaques were neither cleared, nor reduced in size. Several studies using cross-sectional analysis of tissue after chronic treatment of APP mice with γ-secretase inhibitors have demonstrated a reduction in plaque burden that probably reflects prevention of new plaque formation with no effect on plaque sizes [19,36,37]. These data support the idea that γ-secretase inhibition limits the production of new Aβ and therefore reduces the number of new senile plaques deposited without a major effect on the plaques already formed. Together, these data suggest that although γ-secretase inhibition can successfully reduce Aβ production and aggregation these compounds have a limited effect on reversing or clearing Aβ deposits. These data are in accord with experiments performed in a mouse model with inducible APP expression. Nearly complete suppression of the transgene with doxycycline for an additional 6 months after plaque formation still revealed significant amyloid pathology in the brain, suggesting that established plaques do not spontaneously resolve even in the absence of continued Aβ production [38].

There is compelling evidence associating compact senile plaques with synaptic loss and neuritic dystrophy formation [6,30]. Senile plaques also seem to be responsible for the abnormal curvature of the surrounding neurites [4,5,39], disruption of cortical synaptic integration [7] and abnormal calcium regulation in close proximity to senile plaques [40]. It has also been shown that soluble Aβ can provoke cell death by a progressive degeneration that begins in neurites and axons[41]. Taking these considerations into account, we extended our *in vivo *observations to neuronal processes and we assessed the effect of LY-411575 on neuritic abnormalities using multiphoton microscopy. This approach allowed us to explore the possibility of reversing neuritic abnormalities that develop within close proximity of senile plaques. As there is no overt neuronal loss in these mouse models, the neuritic alterations reflect the first steps in neurodegeneration and are a quantifiable index of neuropathology. We observed no effect on the size of neuritic dystrophies closely associated with plaques. Previous studies have shown that these dystrophies can be reduced or eliminated after antibody treatment against Aβ [30] or treatment with curcumin [27]. While the ultimate effect of these dystrophies in the brain is unknown, it is clear that they represent a pathological response to plaques and are not common in healthy tissue.

When we assessed the effect of LY-411575 on neuritic curvature, we observed no effect during the treatment. Neurites are more distorted or "curvy" in the immediate surround of senile plaques, and this curvature can be at least partially reversed with immunotherapy [6] or antioxidant treatment [5,27]. The lack of an effect on the curvature ratio suggests that inhibiting γ secretase does not prevent the neurotoxicity of existing amyloid deposits. It is, however, possible that longer or more potent treatments could prove beneficial.

## Conclusion

All together, our data suggest that the γ-secretase inhibitor LY-411575 can successfully reduce Aβ plasma and brain levels in treated mice, but has no effect over a 3 week period on established amyloid deposits or the neuronal abnormalities associated with senile plaques. This demonstrates that therapeutics aimed at inhibition of Aβ generation are less effective for reversal of existing plaques than for prevention of new plaque formation [19]. Given the relatively short treatment duration of our study, however, longer term studies are warranted to determine whether or not long-term γ-secretase inhibition can reverse the neuropathological abnormalities seen in Alzheimer's disease. Since the ultimate goal would be to restore cognitive impairments that may result from Aβ deposition followed by the associated neurodegeneration, a combination therapy may be warranted. Suppression of Aβ generation along with other therapeutic approaches, including immunotherapy or antioxidant agents that increase clearance of amyloid and reduce the toxic effects of Aβ deposition, could provide a more successful approach to treat the illness.

## Methods

### Animals

Animals were crosses of APPswe/PS1dE9 mice [42] with the YFP expressing mice (thy-1:YFP line H^+/- ^Tg mice [28]) 10–11 months old, obtained from Jackson Lab (Bar Harbor, Maine). All studies were conducted with approved protocols from the Massachusetts General Hospital Animal Care and Use Committee and in compliance with NIH guidelines for the use of experimental animals.

### Reagents

Texas Red dextran 70,000 D was obtained from Molecular probes (Eugene, OR), methoxy-XO4 was a gift from Dr. Klunk (U. Pittsburgh). LY-411575 was synthesized as described [43], and tested for potency using cell based assays [44]. Common chemical reagents where obtained from Sigma (St. Louis, MO).

### *In vivo *treatment and multiphoton imaging

The chronic treatment of APPswe/PS1dE9xYFP involved daily gavage administration of LY-411575 (5 mg/Kg) for 3 weeks. Control animals followed similar procedures but received vehicle instead of γ-secretase inhibitor treatment.

Cranial window surgeries were performed as previously described [45]. Briefly, animals were anesthetized using isoflurane or avertin, the skin and periosteum were removed and a 6-mm diameter craniotomy was performed, making the anterior end immediately anterior to Bregma and the posterior end just anterior to Lambda. Glass windows (8 mm) were installed and secured with dental cement. All animals received an i.p injection of methoxy-XO4 (~2.5 mg/kg), a fluorescent compound that crosses the blood-brain barrier and binds amyloid plaques [46], the day before the surgery. To facilitate finding the same sites in the brain between sessions, Texas Red dextran (70,000 Da, 62.5 mg/kg in sterile PBS) was injected into a lateral tail vein to provide a fluorescent angiogram before every imaging session. As previously described [47] two-photon fluorescence was generated with 800 nm excitation from a mode-locked Ti:Sapphire laser (MaiTai, Spectra-Physics, Mountain View, CA mounted on a multiphoton imaging system (Bio-Rad 1024ES, Bio-Rad, Hercules, CA). A custom-built external detector containing three photomultiplier tubes (Hamamatsu Photonics, Bridgewater, NJ) collected emitted light in the range 380–480, 500–540 and 560–650 nm. 3-color images were acquired for plaques, neurites, and angiography simultaneously using a 20× objective (NA = 0.95, Olympus). *In vivo *images at low resolution (615 × 615 μm; z-step, 5 μm, depth, ~200 μm) were acquired to provide a map of the area, using the angiogram as a 3-D fiducial. LY-411575 treated animals were imaged before the commencement of the treatment (session 1, day 0) and reimaged on a weekly basis for the next 3 consecutive weeks (completing sessions 2, 3 and 4). Control treated animals followed the identical imaging schedule.

### Image processing

Plaque size was measured using the blue fluorescence channel corresponding to methoxy-XO4 labelling. The maximum intensity projection images were thresholded, segmented, and measured using ImageJ software. The same identified plaques were measured from each imaging session such that each plaque served as its own control. The size of each plaque was normalized to 100% at day 0 and then all plaque measurements over time were averaged for summary statistics.

To analyze neurite abnormalities higher resolution, images were captured to identify single neurites and plaques (125 × 125 μm; z-step, 0.8 μm, depth, 20 μm approximately). To exclude motion artifacts induced by heartbeat and breathing, image stacks were aligned using AutoDeblur software (AutoQuant). Images from the green channel (YFP neurites) were further processed with the blind 3D deconvolution function in AutoDeblur to remove background noise. 2D projections of stacks from the three channels were combined in Adobe Photoshop 7 (Adobe Systems). Stacks were used to measure plaque size, dystrophy size, neurite curvature and neurite diameter. Neuritic dystrophies, defined as the areas of swelling in the immediate surrounding of the senile plaques (up to 15 μm from plaques border) [30,48] were outlined on the 2D projections and the areas (in μm^2^) were measured with Image-J software. We also measured as many neurites as we could confidently follow (that were at least 20 μm long) that were present and identifiable in each of the weekly imaging sessions. Thus, the curvature data represent longitudinal imaging with each neurite serving as its own control. The neurite curvature ratio was calculated by dividing the end-to-end distance of a neurite segment by the total length between the two segment ends as previously described [4,6,39]. Neurite shaft diameters were measured at each end and the midpoint of each segment to provide an average diameter along its length. To determine the effect of proximity to plaques, the average distance between the nearest methoxy-XO4 stained amyloid plaque and each neuritic segment was calculated using the average of the distance from the plaque edge to each end and the midpoint of the neuritic segment on the three-channel images, and only neurites located in the proximity of a senile plaque (within 50 μm from a plaque border) were included in the study.

### ELISA measurements

Aβ 40 and 42 were quantified in plasma and brain samples using colorimetric human A-beta 40 and A-beta 42 ELISA kits (WAKO Chemicals USA) as previously described [49] with modifications. Plasma samples were obtained on a weekly basis from the saphenous vein at the end of each imaging session. Blood samples were collected in Eppendorf tubes treated with 10 μl of EDTA (10 mg/ml) and centrifuged at 3500 rpm for 7 minutes. Plasma was frozen at -80 C until the ELISA was run. At the end of the experiments the mice were killed, the brains hemisected and soluble and insoluble Aβ40 and Aβ42 were quantified in flash frozen homogenized hemibrains. For plasma ELISA, samples were diluted in phosphate buffer with 0.2% BSA, 0.4 M NaCl, 0.076% CHAPS, and 2 mM Na2EDTA. Plasma samples were analyzed in duplicate. For brain tissue ELISA, hemibrains were homogenized for 45 s at speed 20 (BioSpec Tissue-Tearor™) in extraction buffer (10 uL/mg brain mass) with protease inhibitor (Complete Protease Cocktail, Roche Diagnostics GmbH, Mannheim, Germany). Extraction buffer consisted of deionized water with 50 mM Tris HCl, 2 mM EDTA 2Na, 0.01% Methiolate Na, 400 mM NaCl, and 1%BSA. One millilitre of each homogenized brain was centrifuged at 15,000 RPM for 5 minutes at 4°C. The supernatant was removed (soluble Aβ, 1:10 final dilution), and the pellet was diluted 1:8 and homogenized in 70% formic acid (800 uL FA for a 100 mg pellet) and centrifuged at 15,000 RPM for 5 minutes at 4°C. Supernatant was removed again (insoluble Aβ) and neutralized in Tris buffer with pH = 11 (1 M Tris with 70% formic acid). The insoluble fraction was further diluted for Aβ 42 measurements. Brain samples were analyzed in triplicate. Standard curves for both plasma and brain tissue ELISAs were made using human Aβ40 and Aβ42 standards provided in the ELISA kit. Absorbance was measured with a Wallac Victor 2 1420 Multilabel Counter (PerkinElmer Life & Analytical Sciences, Shelton, CT) and data were expressed as pmol/g wet tissue.

### Statistical analysis

To assess the dynamics of senile plaque size, neurite curvature, and plasma Aβ levels, two-way ANOVA for repeated measures were used. Dystrophy size, neurite diameter and Aβ brain levels were assessed with one-way ANOVA.

## Competing interests

The authors declare that they have no competing interests.

## Authors' contributions

MS and BJB designed the study. MS carried out the invivio imaging experiments. MG-A and DT analyzed the imaging data. LAB performed the ELISA measurements. AF, PD and TEG characterized and provided LY-411575. TEG and BTH gave critical advice. MG-A and BJB wrote the manuscript. All authors read and approved the final mansucript.
